# Continuous nerve block versus thoracic epidural analgesia for post-operative pain of pectus excavatum repair: a systematic review and meta-analysis

**DOI:** 10.1186/s12871-023-02221-x

**Published:** 2023-08-09

**Authors:** Li-Jung Chen, Shih-Hong Chen, Yung-Lin Hsieh, Po-Chuan Yu

**Affiliations:** Department of Anesthesiology, Taipei Tzu Chi Hospital, Buddhist Tzu Chi Medical Foundation, No.289, Jianguo Rd., Xindian Dist, 231405 New Taipei City, Taiwan

**Keywords:** Pectus excavatum, Thoracic epidural analgesia, Paravertebral block, Erector spinae plane block

## Abstract

Surgery to repair pectus excavatum (PE) is often associated with severe postoperative pain, which can impact the length of hospital stay (LOS). While thoracic epidural analgesia (TEA) has traditionally been used for pain management in PE, its placement can sometimes result in severe neurological complications. Recently, paravertebral block (PVB) and erector spinae plane block (ESPB) have been recommended for many other chest and abdominal surgeries. However, due to the more severe and prolonged pain associated with PE repair, it is still unclear whether continuous administration of these blocks is as effective as TEA. Therefore, we conducted this systematic review and meta-analysis to demonstrate the equivalence of continuous PVB and ESPB to TEA.

**Supplementary Information:**

The online version contains supplementary material available at 10.1186/s12871-023-02221-x.

## Background

Pectus excavatum (PE) is a chest wall deformity [1] that not only affects appearance but may also limit cardiac and pulmonary functions [2, 3]. There are two primary procedures to repair this condition: the Ravitch procedure, which involves a sternal wedge osteotomy, and the Nuss procedure, a minimally invasive approach that involves inserting a metal bar under the sternum, are performed. However, both of these procedures can result in severe pain [4, 5]. Thoracic epidural analgesia (TEA) has a better analgesia effect than intravenous patient-controlled analgesia (PCA) and has been advocated for post-operative PE pain [6]. This procedure involves the placement of either a needle (single injection) or a catheter (continuous infusion) into the epidural space via landmark-guiding, sonography, or fluoroscopy. Subsequently, the solution blocks the ventral and dorsal nerve roots passing through the epidural space [7]. In general terms, TEA catheter implants are considered safe; however, this procedure can lead to severe complications, including spinal cord injury, epidural hematoma, subarachnoid block and epidural abscess [8–10]. Multiple studies [9, 11] have reported chronic and severe neurological complications associated with the use of TEA during the Nuss procedure. Consequently, these adverse outcomes have raised concerns about the safety of TEA.

Other alternatives to TEA, such as paravertebral block (PVB) [11] and erector spinae plane block (ESPB) [12, 13] are also used for post-operative pain management. PVB is a type of peripheral nerve block in which a local anesthetic, with or without other medication, is injected into the paravertebral space. This technique aims to anesthetize the spinal nerves at the paravertebral space, which is immediately lateral to the intervertebral foramina [14]. This produced an ipsilateral segmental somatic block and provides analgesia for chest and abdomen surgeries. The single PVB injection of 15–20 ml or 0.25ml/kg cause unilateral somatic block over 5 dermatomes and sympathetic block over 8 dermatomes [15]. The PVB catheters are usually placed at T4-T6 bilaterally for PE repair. The PVB doses for PE repair are initial 10–20 ml or 0.3–0.6 ml/kg and continous infusion at rate of 5-10ml/hr or 0.125–0.25 ml/kg/hr per catheter [16–18]. The common advantages of PVB compared to TEA are simple, easy to learn, less sympathetic blockade leading to less hypotension and lower risk of urine retension [19]. The similar risks are deep bleeding if coagulopathy, inadvertent vascular, pleural or dural puncture, horner’s syndrome and local anesthetic systemic toxicity [15].

ESPB is another type of peripheral nerve block that involves a local anesthetic, with or without other medication, into erector spinae plane space. The anesthetic diffuses into the paravertebral space anteriorly, working similarly to PVB. An interfascial spread that blocks the posterior rami of spinal nerves may also be another mechanism [20]. The single ESPB injection at T4-6 of 20–30 ml cause sensory block ranging from T1 to L3 [21]. Radiological investigations report that the 2.5 ml local anesthetic solution cover one thoracic dermatome [22]. The ESPB catheters are also placed bilaterally at T4-T6 for PE repair [23–25]. The suggestive ESPB dose for PE repair is 10–20 ml or 0.4ml/kg for loading and continous infusion at rate of 6-12ml/hr. The benefits of ESPB are no sympathectomy if absence of epidural or paravertebral spread, lower risk of urine retension, lower bleeding risk in patients with coagulopathy, no risk for respiratory compression and outpatient ambulatory pump [23–25]. The similar risks are inadvertent vascular or pleural puncture and local anesthetic systemic toxicity. Sensory block was might be in ventral and dorsal dermatomes with variation among studies but mainly posterior to the midaxillary line and minimally to anterior side [26, 27]. Both PVB and ESPB are typically performed under sonography and can be administered as a single injection or continuous infusion. Due to their longer distance from the spinal cord, PVB and ESPB are less likely to cause neurological injury [16, 17, 23] and have been used in numerous thoracic and abdominal surgeries [15, 28, 29]. Several studies have reported their effectiveness and safety; however, there has been no systematic review or meta-analysis regarding the use of nerve blocks in PE repairs. Therefore, this study aims to support the hypothesis that continuous nerve block is equivalent to TEA in managing pain after PE repair.

## Methods

### Study design

**Fig. 1:**
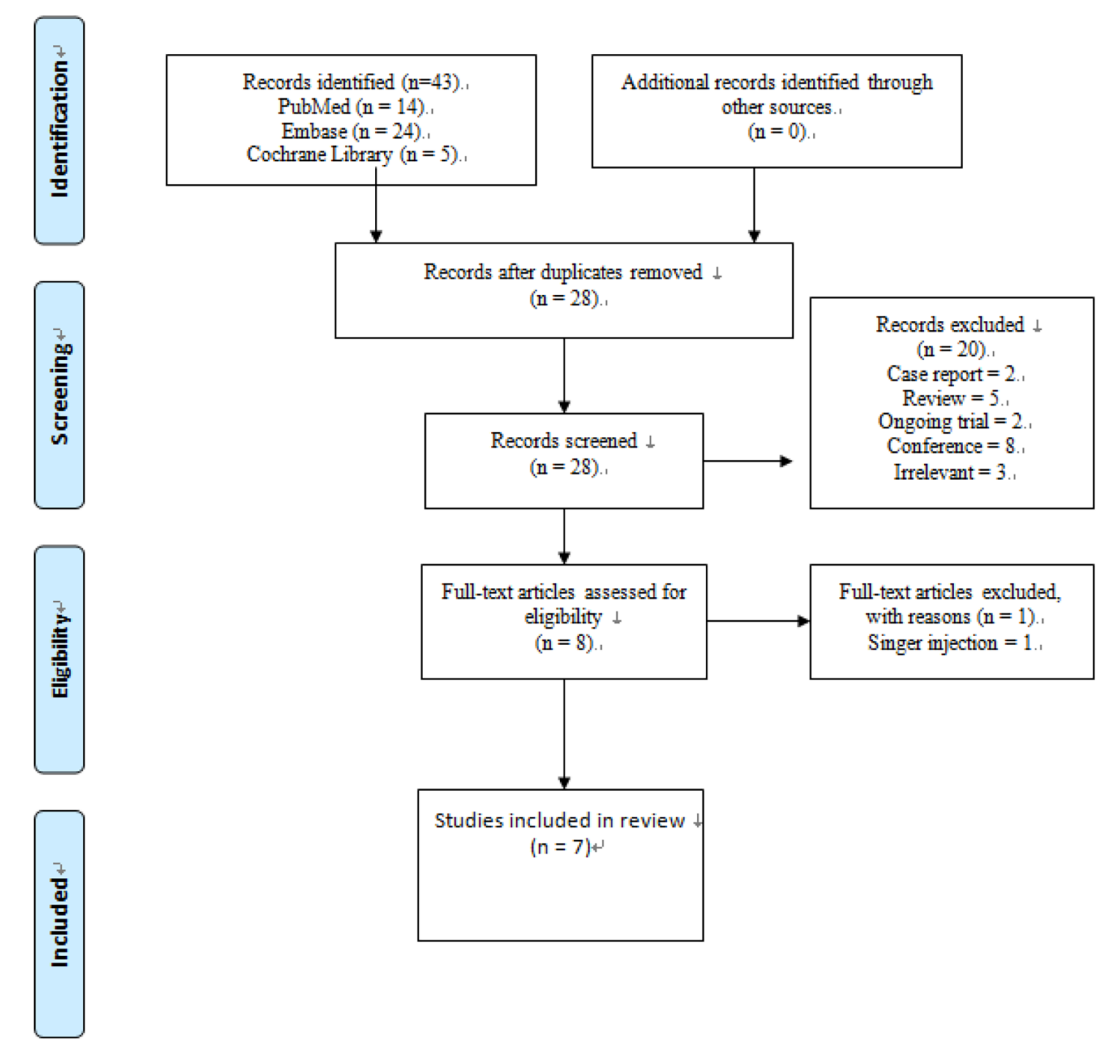
PRISMA flow diagram

This meta-analysis aimed to assess the effects of continuous PVB and ESPB versus TEA on LOS as primary outcome in patients with PE repair. Our study was conducted in accordance with PRISMA guidelines, a commonly used tool to correctly elaborating reviews that evaluate the effects of interventions [30] (Fig. 1).

### Inclusion criteria

The inclusion criteria for studies were as follows: the patients underwent the repair of PE using sterna wedge osteotomy or minimally invasive approach, regardless of the sex, race, age, height, and weight and studies that compared LOS, pain score, and opioid usage between TEA and continuous infusion of ESPB or PVB; both retrospective and prospective studies were eligible.

### Search and selection

PubMed, EMBASE, and Cochrane Library databases were searched for the eligible studies published from their inception until January 2023. We did not exclude studies by date, region, or language. MeSH terms including “Nerve Block/pharmacology“[Mesh], “Nerve Block/standards“[Mesh], “Nerve Block/therapeutic use“[Mesh], “Nerve Block/therapy“[Mesh], “Analgesia, Epidural“[Mesh], “Analgesia, Epidural“[Mesh], “Analgesia, Epidural/adverse effects“[Mesh], “Analgesia, Epidural/statistics and numerical data“[Mesh], “Analgesia, Epidural/therapeutic use“[Mesh], “Analgesia, Epidural/therapy“[Mesh]), “Funnel Chest/surgery“[Mesh], “Funnel Chest/therapy“[Mesh], “Funnel Chest“[Mesh], “Pain Measurement“[Mesh], “Visual Analog Scale“[Mesh], “Analgesics, Opioid“[Mesh], “Analgesics, Opioid/administration and dosage“[Mesh], “Analgesics, Opioid/therapeutic use“[Mesh] and “Length of stay“[Mesh] were used in combination with plain text to search PubMed. Similar strategies were applied to search the other databases. The search strategies are provided in detail in Supplement 1.

Two independent reviewers selected the eligible studies. Any disagreements were resolved by the reviewers; a third reviewer was consulted if the reviewers cannot reach an agreement.

### Data collection

Data extracted from each study included: (i) general study characteristics: study design, country, and enrolment period; (ii) study population characteristics: age, preoperative PE severity index (e.g., Haller index [HI]); (iii) characteristics of the intervention: the level of epidural catheters or continued nerve block catheters that were placed and the medication used for TEA or continued nerve block; (iv) primary outcome measure was LOS; (v) Secondary outcome measures were post-operative pain scores, total opioid usage, and post-operative nausea and vomiting (PONV).

One reviewer extracted data sets from each eligible study, which were further validated by a second reviewer. Continuous variables reported as mean and standard deviation (SD) were extracted without any changes. If needed, the values were driven from the graphs. However, variables reported as median and interquartile range were converted before extraction. Methods of conversion have been reported before [31].

### Risk of bias

The methodological quality of the randomized controlled trial (RCT) was evaluated using the updated Cochrane risk-of-bias tool for randomized trials (RoB 2) [32]. The risk of bias was assessed using the RoB 2 tool, which evaluates the risk-of-bias across five domains and provides a judgment on a 5-point scale. Non-randomized studies were assessed using the Cochrane risk-of-bias tool for non-randomized studies of interventions (ROBINS-I), which assigns studies a rating of low, moderate or high risk (3-point scale) based on seven domains [33].

### Statistics

Meta-analyses were performed by Review Manager Software (version 5.3; The Nordic Cochrane Centre, The Cochrane Collaboration, Copenhagen, Denmark) using a random-effects model. This model was chosen because the treatment effect can vary across each study due to differences in treatment protocols. The primary and secondary outcomes were estimated by the mean difference (MD) and its 95% confidence interval (CI). Statistical heterogeneity was assessed by the Cochran Q statistic and quantified by the *I*^*2*^statistic. Outcomes are presented in forest plots. A subgroup analysis was not predetermined. A P-value ≤ 0.05 was considered statistically significant.

## Results

### Study selection

A total of 43 studies were identified from the three databases, PubMed (n = 14), EMBASE (n = 24), and Cochrane (n = 5). Of them, 15 duplicates were removed. The title and abstract of the remaining 28 studies were screened for eligibility. Twenty studies were excluded due to being irrelevant, case reports, or conference abstracts, among other reasons listed in Fig. 1. The full text of the eight remaining articles was read and thoroughly assessed for eligibility. However, one article was excluded because the patients in both groups had single injections, not continued infusion. The remaining seven studies [16–18, 23–25, 34] were considered eligible for pooling by quantitative synthesis.

### Study characteristics

#### Methods

Among the studies, six were retrospective cohorts [16, 17, 23–25, 34] and one was a prospective observational multi-institutional study [18]. No RCT was found. A total of 644 patients were included. A study did not mention the mean age of participants [34], but it ranged from 14.5 to 15.8 years. The mean of HI was not reported in studies by Loftus et al. [34] and Hall et al.[16], but it ranged from 4.2 to 7.3. Most participants underwent minimally invasive repair of the PE by the Nuss procedure, except 15 participants who underwent the Ravitch procedure in the study by Loftus et al. [34]. Continuous PVB was provided in four studies [16–18, 34]. The patients in three studies received analgesia via ESPB catheter [23–25].

### Interventions

Two studies [16, 24] reported the needle used in the nerve block group: 18G Pajunk needle and 20G B. Braun catheter. The location of TEA catheter placement was not mentioned in one study [34] and mainly reported at T4-T7 in other studies. All the studies except the one by Loftus et al. [34] did not report the formulation, bupivacaine or ropivacaine with clonidine or dexmedetomidine, of continuous peripheral nerve block infusion. Additional PCA pumps were administered in one study [17]. Three studies [16, 17, 24] reported the location of TEA catheter placement, which was mostly between T4-5, T5-6, or T6-7. In studies with mention, the epidural space was infused with bupivacaine or ropivacaine in combination with an opioid. Main characteristics of these studies were listed in Table 1.

### Outcomes

Five studies reported the LOS [18, 23–25, 34]. Pain score (mostly measured using NRS in those studies describing it), opioid usage, and events of PONV were at least reported by one study each. A summary of main outcomes is provided in Table 2.

**Table 1:** Study characteristics

Study	Nation		Study design	Treatment arm	Period	Number of patients	Age (years)	Level of catheter	Medications	HI
Hall 2014 [16]	USA		Retrospective case-control study	PVB	2009/06– 2011/08	10	15.5 ± 2.3	T6 TP	7ml/hr 0.2% R	NR
				TEA	2010/10– 2012/01	10	14.5 ± 2.5	T5	7ml/hr 0.2% R	NR
Loftus 2016 [34]	USA		Retrospective observation study	PVB	2009/01– 2012/12	28	NR	NR	NR	NR
				TEA		80	NR	NR	NR	NR
Beltran 2017 [17]	Canada		Retrospective observation study	PVB	2011– 2013	7	15.7 ± 1.3	T45,T56	0.25–0.5 mg/kg 0.2% R with PCA	4.8 ± 1.6
				TEA		8	15.8 ± 1.6	T567	0.1% R or 0.125% B with fentanyl 2mcg/ml	4.5 ± 1.4
Muhly 2019 [18]	USA		Prospective observation multi-institutional study	PVB	2014/6– 2015/8	56	14.9 ± 2.8	D	D	4.8 ± 1.6
				TEA		114	14.9 ± 2.4	D	D	4.5 ± 1.4
Bliss 2022 [25]	USA		Retrospective observation study	ESP	NR	30	15.4 ± 1.2	T5–6	0.5% R 6ml/hrinitial (0.25 mg/kg/hr max)	7.3 ± 1.6
				TEA		30	14.9 ± 1.3	NR	0.2% R with hydromorphine 2–5mcg/ml	4.2 ± 1.4
Santana 2022 [23]	USA		Retrospective observation study	ESP	2014/1– 2020/1	19	15.6 ± 1.8	T4–T6	0.2% R with 1mcg/ml clonidine	5.2 ± 1.1
				TEA		41	15 ± 2.2	NA	NR	5.5 ± 2.2
Walter 2023 [24]	USA		Retrospective observation study	ESP	2019/1– 2021/5	97	15.3 ± 2.3	T5 TP	0.125–0.2% R 6–8ml/hr with clonidine 0.5mcg/ml	4.8 ± 4.6
				TEA		114	15 ± 3	T45T56	0.2% R 10–12ml/hr	4.9 ± 4.6

TP: transverse process; R: ropivacaine; NR: not reported; B: bupivacaine; D: differs in each hospital

**Table 2:** Study outcomes

Study	Treatment arm	LOS (days)	Time to remove analgesia catheter (days)	Pain Score on POD 1 POD 2 POD 3	Opioid usage (mg/kg) on POD 1 POD 2 POD 3	PONV
Hall 2014 [16]	PVB	NR	3	3.367 ± 1.548 1.667 ± 2.752 2.667 ± 2.408	0.14 ± 0.12 0.14 ± 0.1 0.14 ± 0.1	NR
	TEA	NR	3	3.133 ± 1.118 2.833 ± 2.58 3.167 ± 2.408	0.157 ± 0.181 0.163 ± 0.138 0.163 ± 0.172	NR
Loftus 2016 [34]	PVB	3.3 ± 2.339	NR	3.523 ± 4.7 3.113 ± 4.421 2.893 ± 4	NR	NR
	TEA	5.3 ± 3.775	NR	3.3 ± 5.662 3.267 ± 5.36 2.93 ± 4.462	NR	NR
Beltran 2017 [17]	PVB	NR	NR	2.5 ± 1.4 2 ± 1.1 1.6 ± 1.2	3.4 ± 1.6 3.4 ± 2 3.2 ± 1.5	2
	TEA	NR	NR	2.2 ± 1.6 2.7 ± 2.4 2.5 ± 2.4	1.4 ± 1.4 2 ± 2 3.1 ± 2.3	4
Muhly 2019 [18]	PVB	3 ± 1.52	D	3.333 ± 2.282 3 ± 1.522 3 ± 1.522	0.31 ± 0.175 0.33 ± 0.19 0.08 ± 0.167	13
	TEA	4 ± 1.5	D	2.33 ± 2.252 2.33 ± 2.252 2.33 ± 2.252	0.0066 ± 0.015 0.0166 ± 0.037 0.06 ± 0.112	45
Bliss 2022 [25]	ESP	2.9 ± 0.87	5 ± 1.34	3.9 ± 1.82 3.97 ± 1.82 4.32 ± 2.53	1 ± 0.575 0.6785 ± 0.3875 0.7125 ± 0.4964	NR
	TEA	3.78 ± 0.82	2.84 ± 0.3	2.72 ± 1.37 2.83 ± 1.32 3.38 ± 1.67	0.2125 ± 0.0875 0.2625 ± 0.1267 0.8339 ± 0.366	NR
Santana 2022 [23]	ESP	3.3 ± 0.5	NR	4.1 ± 1.682 4.067 ± 1.282 4.067 ± 1.442	NR	NR
	TEA	4.7 ± 0.9	NR	2.667 ± 0.922 3 ± 0.307 3.333 ± 0.922	NR	NR
Walter 2023 [24]	ESP	2 ± 0	POD 5	4.48 ± 1.36	0.1 ± 0.0978 0.0167 ± 0.0376 0 ± 0	4
	TEA	3.33 ± 0.75	POD 2	3.83 ± 1.59	0.28 ± 0.06 0.013 ± 0.03 0.02 ± 0.045	33

### Risk of bias

**Fig. 2:**
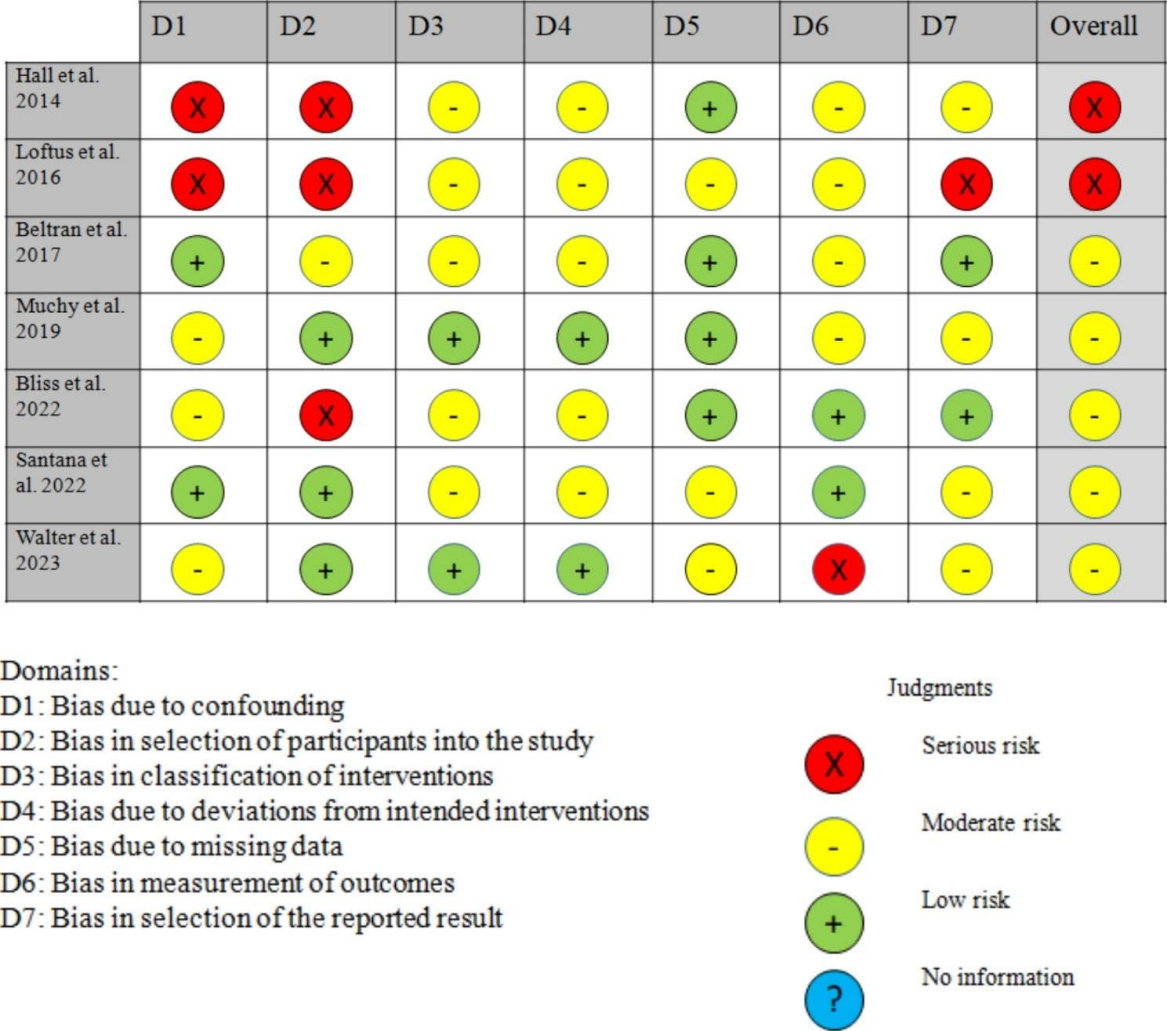
Risk of bias within studies

Two studies [16, 34] were assessed as having a serious risk of bias, while five studies were judged as having a moderate risk [17, 18, 23–25] (see Fig. 2).

### Length of hospital stay

**Fig. 3:**
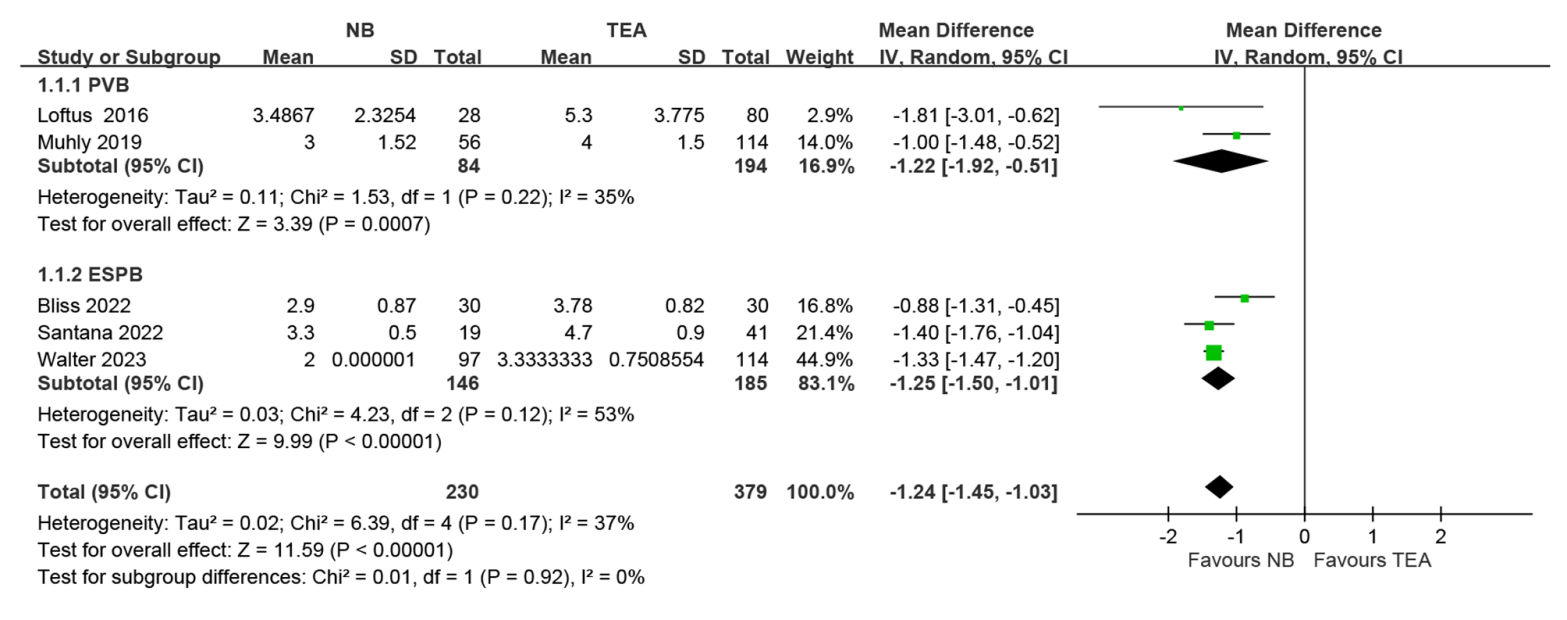
Meta-analyses of the LOS

Five studies reported LOS [18, 23–25, 34]. The pooled effect estimate showed a significantly reduced LOS in the nerve block group than in the TEA group (Fig. 3; MD, -1.24; 95% CI, -1.45 to -1.03; P < 0.001). No significant between-subgroup differences (*I²* = 0%; P = 0.92) were observed. (PVB; MD -1.22, 95% CI, -1.92 to -0.51; P < 0.001; ESPB; MD -1.25, 95% CI, -1.5 to -1.04; P < 0.001)

### Pain score

**Fig. 4:**
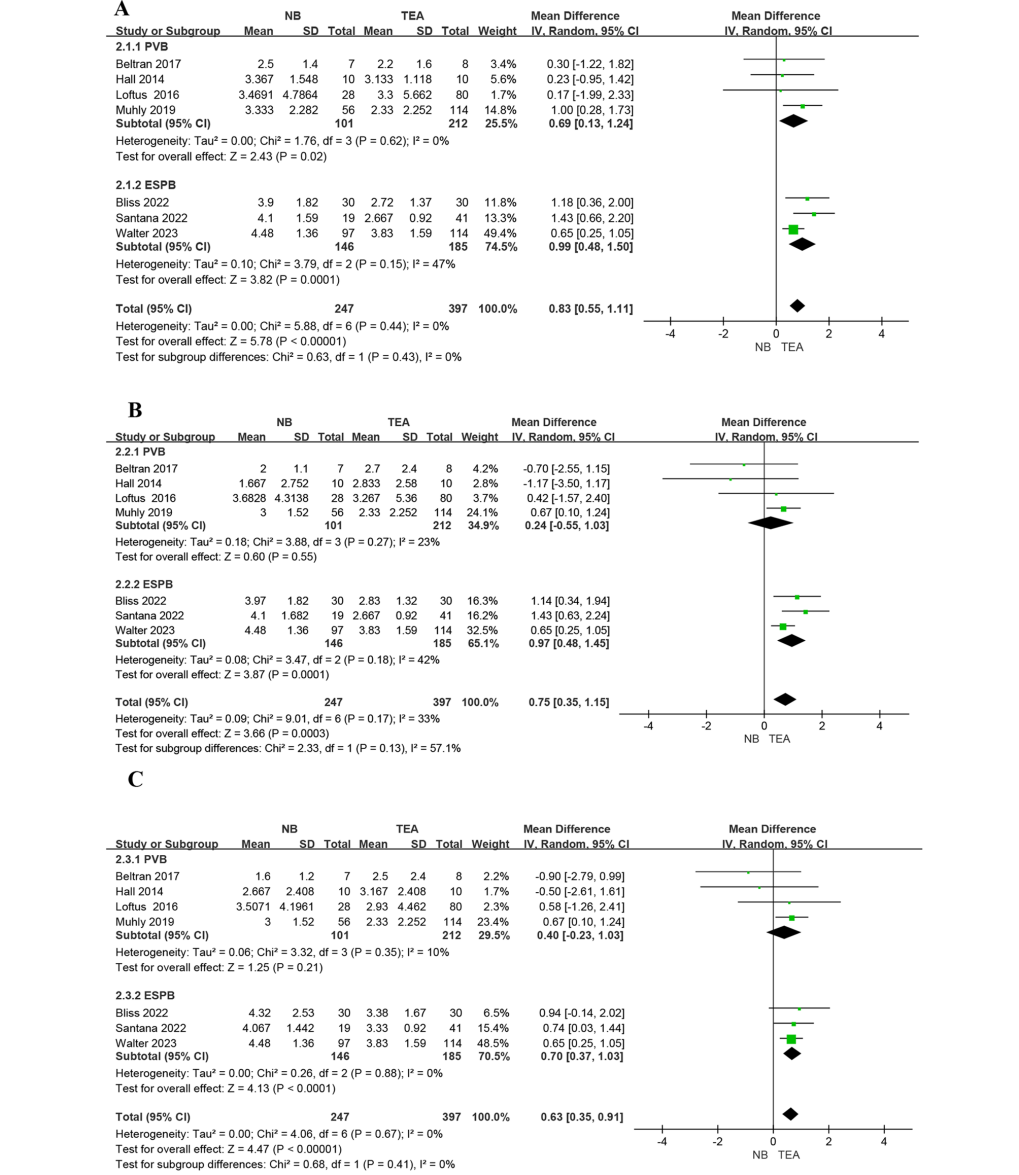
Forest plot of pain score on POD 1 **(A)**, POD 2 **(B)** and POD 3 **(C)**

All the studies reported pain scores. The synthesis showed a significantly lower pain score in the TEA group by 0.83 (95% CI, 0.55 to 1.11; P < 0.001), 0.75 (95% CI, 0.35 to 1.15; P < 0.001), and 0.63 (95% CI, 0.35 to 0.91; P < 0.001) on post-operative days (POD) 1, 2, and 3, respectively (see Fig. 4). No significant between-subgroup differences (*I²* = 0%; P = 0.43) were observed on POD1 (PVB; MD 0.69, 95% CI, 0.13 to 1.24; P = 0.02; ESPB; MD 0.99, 95% CI, 0.48 to 1.5; P < 0.001). There was an insignificant between-subgroup difference (*I²* = 57.1%; P = 0.13) on POD2 and the difference between PVB and TEA was insignificant while TEA group has a lower pain score than ESPB group (PVB; MD 0.24, 95% CI, -0.55 to 1.03; P = 0.55; ESPB; MD 0.97, 95% CI, 0.48 to 1.45; P < 0.001). There was no significant between-subgroup difference (*I²* = 0%; P = 0.41) on POD3 and the difference between PVB and TEA was also insignificant while TEA group still has a lower pain score than ESPB group (PVB; MD 0.40, 95% CI, -0.23 to 1.03; P = 0.21; ESPB; MD 0.63, 95% CI, 0.35 to 0.91; P < 0.001).

### Opioid usage

**Fig. 5:**
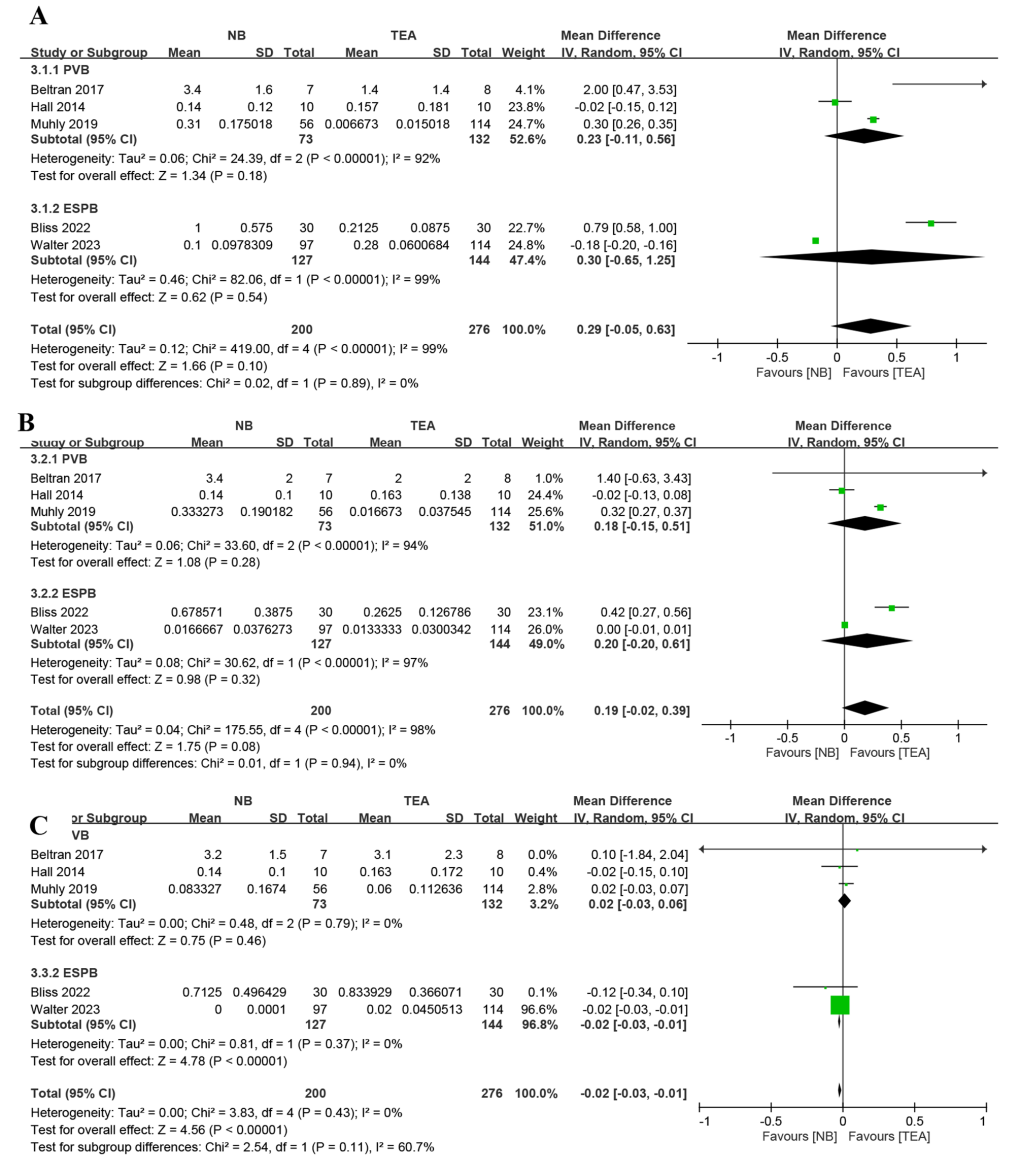
Forest plot of opioid usage on POD 1 **(A)**, POD 2 **(B)** and POD 3 **(C)**

Five studies reported opioid usage on post-operative day 1 to day 3 [16–18, 24, 25]. The meta-analysis showed no significant difference on post-operative day 1 (MD 0.29, 95% CI, -0.05 to 0.63; P = 0.10) and 2 (MD 0.19, 95% CI, -0.02 to 0.39; P = 0.08) but significantly high opioid usage in TEA group on post-operative day 3 (Fig. 5; MD − 0.02, 95% CI, -0.03 to -0.01; P < 0.001). No significant between-subgroup differences were observed on POD1 (*I²* = 0%; P = 0.89, PVB; MD 0.23, 95% CI, -0.11 to 0.56; P = 0.18; ESPB; MD 0.3, 95% CI, -0.65 to 1.25; P = 0.54), on POD2 (*I²* = 0%; P = 0.94, PVB; MD 0.18, 95% CI, -0.15 to 0.51; P = 0.28; ESPB; MD 0.2, 95% CI, -0.2 to 0.61; P = 0.32) and on POD3 (*I²* = 60.7%; P = 0.11, PVB; MD 0.02, 95% CI, -0.03 to 0.06; P = 0.46; ESPB; MD -0.02, 95% CI, -0.03 to -0.01; P = 0.37). However, due to high-level of heterogeneity in test for subgroup differences on POD3, the heterogeneity decreased to low-level (*I²* = 31.2%; P = 0.23) after we removed the study by Walter [24] and POD3 opioid usage difference became insignificant (MD 0.01, 95% CI, -0.03 to 0.06; P = 0.61).

### Event of post-operative nausea or vomiting

**Fig. 6:**
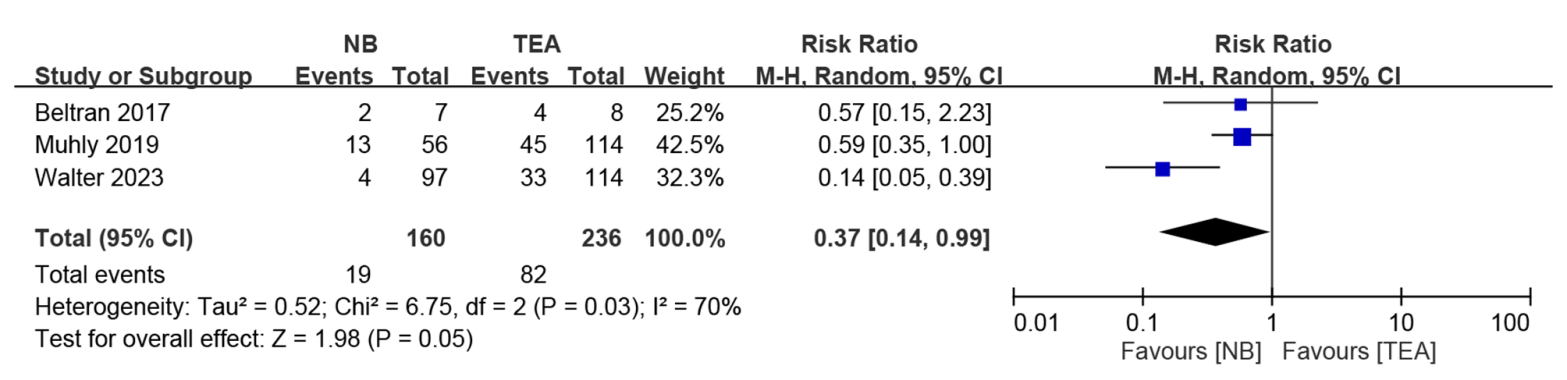
Forest plot of PONV

Three studies reported events of PONV [17, 18, 24]. The analysis showed a significant difference in more events of PONV in TEA group than nerve block group (Fig. 6; Risk ratio 0.37, 95% CI, 0.14 to 0.99; P = 0.05). However, there was a high-level of heterogeneity (*I²* = 70%; P = 0.03).

## Discussion

This systematic review and meta-analysis compared continuous ESPB and PVB with TEA about LOS as the primary outcome. However, no RCT was found in this regard. Seven non-randomized studies were included, with a total of 644 patients. The synthesis of the result reported that continuous nerve blockage reduced LOS by 1.24 days compared to TEA with statistical significance in patient with PE repair (Fig. 3). Several reasons may cause this result. Firstly, the duration of urinary catheter usage was much longer in the TEA group [18, 25]. Recent studies reported around 10% patients who underwent TEA experienced urinary retention [35, 36] due to the effect of epidural mixture on their urethral sphincter function [37]. This can cause difficulties voiding, leading to the need for longer use of indwelling urinary catheters [37]. However, the use of catheters can limit a patient’s ability to move around freely [38]. In other meta-analysis involving patients who underwent thoracotomy, they also reported that urinary retention is more common in TEA group than PVB group [39]. Secondly, patients in the TEA group reported experiencing numbness and weakness in their upper arms, chest, and sometimes even their lower legs [10, 24]. This can result in a decline in ambulation due to reduced muscle power in the limbs, or due to the patient’s unwillingness to move. Thirdly, the use of opioids and PONV also play an important role in determining discharge, which we will discuss further later on. Lastly, TEA provided excellent analgesia, which resulted in patients having a painless experience and making it difficult to discontinue the treatment [25]. This can sometimes lead to rebound pain after the weaning process [23].

Pain scores were significantly lower in TEA group from POD 1 to POD 3 compared to nerve block group [25, 40] (Fig. 4). This is likely due to the excellent efficacy of TEA. In another meta-analysis involving patients who underwent thoracotomy, another painful thoracic surgery traditionally treated with TEA, the TEA group had lower pain scores than the continuous nerve block group in the first 48 h [39]. However, in subgroup analysis, pain score was significantly lower in TEA group than PVB only on POD 1 while pain scores on POD 2 and 3 were not significantly different. This result was also noted in study of Liang et al. [19], which demonstrated the PVB group had higher pain scores in first 1–2 h and 4–6 h but there were no difference on 24 and 48 h postoperatively. While there were statistically significant differences in pain scores in our study and in patients with thoracotomy, these differences were small and not more than 1 point [39]. These small differences can be easily managed by additional oral or parenteral analgesia.

Opioid usage was not significantly different in the first two days after the operation. However, on POD 3, there was a significant difference in opioid usage (Fig. 5). This phenomenon might come from the removal of TEA catheter on POD2 or using clonidine as an adjuvant for ESPB in study of Walter et al. [24] and the request for oral medication due to rebound pain after epidural discontinuation [23]. Santana et al. reported that patients in the TEA group consume more opioids72 hours after the operation. The opioid usage after POD 7 was more frequent in the TEA group [24]. The total opioid usage may be higher in the TEA group duration hospitalization [23, 24]. The postoperative opioid usage was another important factor for LOS [41, 42]. However, due to variations in the duration of analgesia catheter use and insufficient data, we were unable to evaluate the pain and opioid usage after discontinuing both types of analgesia catheters.

Our analysis found a higher risk of PONV needing treatment in the TEA group (Fig. 6) which may relate to the neuraxial administration of opioids [18]. A similar result was reported by Scarci et al. [29] They found that PONV was observed in 35% of patients who received neuraxial opioids without prophylactic antiemetics [43]. PONV can cause a great deal of discomfort for patients and may hinder their ability to consume food orally. In such cases, intravenous fluids may be required for a longer duration due to poor oral intake [44]. Therefore, PONV has been found to be highly correlated with the LOS [45].

Although TEA provides better pain control compared to continuous ESPB and PVB, catheter placement is considered a technical skill [22] and has a relatively high failure rate [29]. Unintentional dural puncture, a risk factor for spinal cord damage, has been reported at a rate of 0.4–3.4% [8]. ESPB [23] and PVB [16, 17] are less likely to cause damage to the spinal cord as they are placed at a greater distance from the neuraxial axis. The current literature has not reported any permanent neurological defects resulting from ESPB [23] or PVB [16]. Other complications related to ESPB [26] and PVB [27], such as pneumothorax, pleural puncture, epidural or intrathecal spread, hematoma, and vascular puncture, have been reported, but at a lower rate compared to TEA. The common complications associated with PVB catheter placement are vascular puncture (3.8%), pleural puncture (1.1%), and pneumothorax (0.5%) [46]. A pooled review of ESPB identified a case of pneumothorax [47]. However, there is a steep learning curve associated with ESP catheter placement [25] and it takes longer (21 min) to place them bilaterally [24, 28]. Overall, continuous nerve blocks appear to be safer than TEA. Complications such as vascular puncture and pneumothorax are easier to manage than neurological damage. Therefore, ESPB and PVB may be considered safer alternatives.

TEA is currently the most common method of pain control for patients undergoing major thoracic and abdominal surgery [11, 48, 49]. However, with the advancement of ultrasound equipment and concerns about neurological injury, more anesthesiologists are using nerve blocks for these patients [50]. Although several studies have demonstrated the efficacy of nerve blocks [4, 39, 40], the pain characteristics of patients undergoing PE repair are different from those of patients undergoing other major thoracic surgeries. These patients seldom felt severe incision pain but often experience stretch and pressure on the sternum and chest wall [18, 51]. Therefore, it may not be appropriate to directly apply findings from other studies to patients undergoing PE repair. Fortunately, our report demonstrated that continuous nerve blocks are non-inferior to TEA in this patient population. However, there were no RCT conducted. Future studies should be well-designed RCTs that compare TEA and continuous nerve blocks in terms of LOS, pain score, and adverse events in patients undergoing PE repair. Moreover, as these patients often experience longer pain durations, studies should also assess pain scores, opioid consumption, and PONV after the weaning of analgesia catheters.

### Limitations

The main limitation of this study is the low number of included studies. In addition, none of the studies were RCTs, and there was a moderate to serious risk of bias in the included trials. Furthermore, the use of data conversion methods may have introduced some limitations to our analysis.

## Conclusion

Continuous PVB and ESPB use is associated with reduced hospital stay and PONV, and thus, may be considered alternative to thoracic epidural analgesia in patients with PE repair. However, because of the low quality of studies, well-designed RCT are required to verify the evidence.

### Electronic supplementary material

Below is the link to the electronic supplementary material.

Supplementary Material 1

## Data Availability

All data generated or analysed during this study are included in this published article.

## Abbreviations

CI: Confidence interval
HI: Haller index
ESPB: Erector spinae plane block
LOS: Length of hospital stay
PVB: Paravertebral block
PCA: Patient-controlled analgesia
PE: Pectus excavatum
POD: Post-operative day
PONV: Post-operative nausea and vomiting
RCT: Randomized controlled trial
ROBINS-I: Risk-of-bias tool for non-randomized studies of interventions
MD: Mean difference
TEA: Thoracic epidural analgesia
