# Supplementary material for: Continuous nerve block versus thoracic epidural analgesia for post-operative pain of pectus excavatum repair: a systematic review and meta-analysis
Source: BMC Anesthesiol. 2023 Aug 9;23:266. doi: 10.1186/s12871-023-02221-x (PMC10410789; doi:10.1186/s12871-023-02221-x)
Supplement: Supplementary file 1 — Supplementary Material 1 [file 12871_2023_2221_MOESM1_ESM.docx]

Supplementary material:

Detail literature search strategies in PubMed, Embasee, and Cochrane Library databases

**PubMed:**

Step 1: "Nerve Block/pharmacology"[Mesh] OR "Nerve Block/standards"[Mesh] OR "Nerve Block/therapeutic use"[Mesh] OR "Nerve Block/therapy"[Mesh] OR "nerve block"[TIAB] OR "plane block"[TIAB] OR "erector spinae plane"[TIAB] OR "erector spinae plane block"[TIAB] OR "thoracic paravertebral"[TIAB] OR "thoracic paravertebral block"[TIAB] OR "paravertebral block"[TIAB] OR "paravertebral"[TIAB] OR "erector spinae plane" OR "erector spinae plane block" OR "thoracic paravertebral" OR "thoracic paravertebral block" OR "paravertebral block" OR "paravertebral"

Step 2: "Analgesia, Epidural"[Mesh] OR "Analgesia, Epidural"[Mesh] OR "Analgesia, Epidural/adverse effects"[Mesh] OR "Analgesia, Epidural/statistics and numerical data"[Mesh] OR "Analgesia, Epidural/therapeutic use"[Mesh] OR "Analgesia, Epidural/therapy"[Mesh] OR "patient-controlled epidural analgesia"[TIAB] OR "patient controlled epidural analgesia"[TIAB] OR "epidural patient-controlled analgesia"[TIAB] OR "patient controlled epidural analgesia"[TIAB] OR "epidural analgesia"[TIAB] OR "epidural"[TIAB] OR "thoracic epidural"[TIAB] OR "thoracic epidural analgesia" [TIAB] OR "patient-controlled epidural analgesia" OR "patient controlled epidural analgesia" OR "epidural patient-controlled analgesia" OR "patient controlled epidural analgesia" OR "epidural analgesia" OR "epidural" OR "thoracic epidural" OR "thoracic epidural analgesia"

Step 3: "Funnel Chest/surgery"[Mesh] OR "Funnel Chest/therapy"[Mesh] OR "Funnel Chest"[Mesh] OR "Minimally invasive pectus excavatum repair"[TIAB] OR "Minimal invasive pectus excavatum repair"[TIAB] OR "Minimal-invasive pectus excavatum repair"[TIAB] OR "pectus excavatum repair"[TIAB] OR "pectus excavatum"[TIAB] OR "minimally invasive repair of pectus excavatum"[TIAB] OR "repair of pectus excavatum"[TIAB] OR "Nuss procedure"[TIAB] OR "Funnel Chest"[TIAB] OR "Funnel Chest repair"[TIAB] OR "repair of Funnel Chest"[TIAB] OR "Minimally invasive pectus excavatum repair" OR "Minimal invasive pectus excavatum repair" OR "Minimal-invasive pectus excavatum repair" OR "pectus excavatum repair" OR "pectus excavatum" OR "minimally invasive repair of pectus excavatum" OR "repair of pectus excavatum" OR "Nuss procedure" OR "Funnel Chest" OR "Funnel Chest repair" OR "repair of Funnel Chest"

Step 4: "Pain Measurement"[Mesh] OR "Visual Analog Scale"[Mesh] OR "Pain score"[TIAB] OR "Numerical rating scale"[TIAB] OR "Visual Analogue Scale"[TIAB] OR "Categorical scale"[TIAB] OR "Descriptor Differential Scale"[TIAB] OR "Mankoski Pain Scale"[TIAB] OR "Children's Revised Impact of Event Scale"[TIAB] OR "McGill Pain Questionnaire"[TIAB] OR "COMFORT Behavior Scale"[TIAB] OR "Visual Analog Scales"[TIAB] OR "Pain Measurement"[TIAB] OR "Pain Measurements"[TIAB] OR "Pain Assessment"[TIAB] OR "Pain Assessments"[TIAB] OR "McGill Pain Scale"[TIAB] OR "McGill Pain Scales"[TIAB] OR "Pain Intensity"[TIAB] OR "Pain Intensities"[TIAB] OR "Pain Severity"[TIAB] OR "Pain Severities"[TIAB] OR "Analogue Pain Scale"[TIAB] OR "Analogue Pain Scales"[TIAB] OR "Analog Pain Scale"[TIAB] OR "Analog Pain Scales"[TIAB] OR "Visual Analog Pain Scale"[TIAB] OR "Visual Analog Pain Scales"[TIAB] OR "Visual Analogue Pain Scale"[TIAB] OR "Visual Analogue Pain Scales"[TIAB] OR "Pain score" OR "Numerical rating scale" OR "Visual Analogue Scale" OR "Categorical scale" OR "Descriptor Differential Scale" OR "Mankoski Pain Scale" OR "Children's Revised Impact of Event Scale" OR "McGill Pain Questionnaire" OR "COMFORT Behavior Scale" OR "Visual Analog Scales" OR "Pain Measurement" OR "Pain Measurements" OR "Pain Assessment" OR "Pain Assessments" OR "McGill Pain Scale" OR "McGill Pain Scales" OR "Pain Intensity" OR "Pain Intensities" OR "Pain Severity" OR "Pain Severities" OR "Analogue Pain Scale" OR "Analogue Pain Scales" OR "Analog Pain Scale" OR "Analog Pain Scales" OR "Visual Analog Pain Scale" OR "Visual Analog Pain Scales" OR "Visual Analogue Pain Scale" OR "Visual Analogue Pain Scales" OR "Analgesics, Opioid"[Mesh] AND "Analgesics, Opioid/administration and dosage"[Mesh] OR "Analgesics, Opioid/therapeutic use"[Mesh] OR "Opioid"[TIAB] OR "Opioid usage"[TIAB] OR "opioid consumption"[TIAB] OR "opioid use"[TIAB] OR "usage of opioid"[TIAB] OR "consumption of opioid"[TIAB] OR "Opioid" OR "Opioid usage" OR "opioid consumption" OR "opioid use" OR "usage of opioid" OR "consumption of opioid" OR "Length of stay"[Mesh] OR "Length of stay"[TIAB] OR "Stay Length"[TIAB] OR "Stay Lengths"[TIAB] OR "Hospital Stay"[TIAB] OR "Hospital Stays"[TIAB] OR "Length of stay" OR "Stay Length" OR "Stay Lengths" OR "Hospital Stay" OR "Hospital Stays"

Step 5: #1 AND #2 AND #3 AND #4

**Embae and Cochrane Library:**

Step 1: "nerve block" OR "plane block" OR "erector spinae plane" OR "erector spinae plane block" OR "thoracic paravertebral" OR "thoracic paravertebral block" OR "paravertebral block" OR "paravertebral" OR "erector spinae plane" OR "erector spinae plane block" OR "thoracic paravertebral" OR "thoracic paravertebral block" OR "paravertebral block" OR "paravertebral"

Step 2: "Analgesia, Epidural" OR "patient-controlled epidural analgesia" OR "patient controlled epidural analgesia" OR "epidural patient-controlled analgesia" OR "patient controlled epidural analgesia" OR "epidural analgesia" OR "epidural" OR "thoracic epidural" OR "thoracic epidural analgesia" OR "patient-controlled epidural analgesia" OR "patient controlled epidural analgesia" OR "epidural patient-controlled analgesia" OR "patient controlled epidural analgesia" OR "epidural analgesia" OR "epidural" OR "thoracic epidural" OR "thoracic epidural analgesia"

Step 3: "Funnel Chest" OR "Minimally invasive pectus excavatum repair" OR "Minimal invasive pectus excavatum repair" OR "Minimal-invasive pectus excavatum repair" OR "pectus excavatum repair" OR "pectus excavatum" OR "minimally invasive repair of pectus excavatum" OR "repair of pectus excavatum" OR "Nuss procedure" OR "Funnel Chest" OR "Funnel Chest repair" OR "repair of Funnel Chest" OR "Minimally invasive pectus excavatum repair" OR "Minimal invasive pectus excavatum repair" OR "Minimal-invasive pectus excavatum repair" OR "pectus excavatum repair" OR "pectus excavatum" OR "minimally invasive repair of pectus excavatum" OR "repair of pectus excavatum" OR "Nuss procedure" OR "Funnel Chest" OR "Funnel Chest repair" OR "repair of Funnel Chest"

Step 4: "Pain Measurement" OR "Visual Analog Scale" OR "Pain score" OR "Numerical rating scale" OR "Visual Analogue Scale" OR "Categorical scale" OR "Descriptor Differential Scale" OR "Mankoski Pain Scale" OR "Children's Revised Impact of Event Scale" OR "McGill Pain Questionnaire" OR "COMFORT Behavior Scale" OR "Visual Analog Scales" OR "Pain Measurement" OR "Pain Measurements" OR "Pain Assessment" OR "Pain Assessments" OR "McGill Pain Scale" OR "McGill Pain Scales" OR "Pain Intensity" OR "Pain Intensities" OR "Pain Severity" OR "Pain Severities" OR "Analogue Pain Scale" OR "Analogue Pain Scales" OR "Analog Pain Scale" OR "Analog Pain Scales" OR "Visual Analog Pain Scale" OR "Visual Analog Pain Scales" OR "Visual Analogue Pain Scale" OR "Visual Analogue Pain Scales" OR "Pain score" OR "Numerical rating scale" OR "Visual Analogue Scale" OR "Categorical scale" OR "Descriptor Differential Scale" OR "Mankoski Pain Scale" OR "Children's Revised Impact of Event Scale" OR "McGill Pain Questionnaire" OR "COMFORT Behavior Scale" OR "Visual Analog Scales" OR "Pain Measurement" OR "Pain Measurements" OR "Pain Assessment" OR "Pain Assessments" OR "McGill Pain Scale" OR "McGill Pain Scales" OR "Pain Intensity" OR "Pain Intensities" OR "Pain Severity" OR "Pain Severities" OR "Analogue Pain Scale" OR "Analogue Pain Scales" OR "Analog Pain Scale" OR "Analog Pain Scales" OR "Visual Analog Pain Scale" OR "Visual Analog Pain Scales" OR "Visual Analogue Pain Scale" OR "Visual Analogue Pain Scales" OR "Analgesics, Opioid" OR "Opioid" OR "Opioid usage" OR "opioid consumption" OR "opioid use" OR "usage of opioid" OR "consumption of opioid" OR "Opioid" OR "Opioid usage" OR "opioid consumption" OR "opioid use" OR "usage of opioid" OR "consumption of opioid" OR "Length of stay" OR "Length of stay" OR "Stay Length" OR "Stay Lengths" OR "Hospital Stay" OR "Hospital Stays" OR "Length of stay" OR "Stay Length" OR "Stay Lengths" OR "Hospital Stay" OR "Hospital Stays"

Step 5: #1 AND #2 AND #3 AND #4
